# MET overexpression in ovarian cancer via CD24‐induced downregulation of miR‐181a: A signalling for cellular quiescence‐like state and chemoresistance in ovarian CSCs

**DOI:** 10.1111/cpr.13582

**Published:** 2023-11-29

**Authors:** Ji Eun Kwon, Yeonsue Jang, Bo Seong Yun, Suki Kang, Yon Hee Kim, Baek Gil Kim, Nam Hoon Cho

**Affiliations:** ^1^ Department of Pathology Ajou University School of Medicine Suwon Korea; ^2^ Brain Korea 21 Plus Project for Medical Science Yonsei University College of Medicine Seoul Korea; ^3^ Department of Pathology Yonsei University College of Medicine Seoul Korea; ^4^ Department of Gynecology Obstetrics and Gynecology, CHA Gangnam Medical Center CHA University Seoul Korea; ^5^ Department of Pathology Soonchunhyang University Hospital Seoul Korea; ^6^ Severance Biomedical Science Institute (SBSI) Yonsei University College of Medicine Seoul Korea

## Abstract

Increased expression of CD24 and MET, markers for cancer stem‐like cells (CSCs), are each associated with ovarian cancer severity. However, whether CD24 and MET are co‐expressed in ovarian CSCs and, if so, how they are related to CSC phenotype manifestation remains unknown. Our immunohistochemistry analysis showed that the co‐expression of CD24 and MET was associated with poorer patient survival in ovarian cancer than those without. In addition, analyses using KM plotter and ROC plotter presented that the overexpression of CD24 or MET in ovarian cancer patients was associated with resistance to platinum‐based chemotherapy. In our miRNA transcriptome and putative target genes analyses, miR‐181a was downregulated in CD24‐high ovarian cancer cells compared to CD24‐low and predicted to bind to CD24 and MET 3'UTRs. In OV90 and SK‐OV‐3 cells, CD24 downregulated miR‐181a expression by Src‐mediated YY1 activation, leading to increased expression of MET. And, CD24 or MET knockdown or miR‐181a overexpression inhibited the manifestation of CSC phenotypes, cellular quiescence‐like state and chemoresistance, in OV90 and SK‐OV‐3 cells: increased colony formation, decreased G0/G1 phase cell population and increased sensitivity to Cisplatin and Carboplatin. Our findings suggest that CD24‐miR‐181a‐MET may consist of a signalling route for ovarian CSCs, therefore being a combinatory set of markers and therapeutic targets for ovarian CSCs.

## Graphical abstract

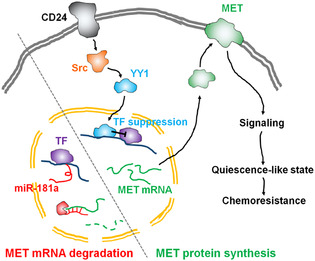

## 1 INTRODUCTION

CD24 and MET expression are associated with cancer malignancy and cancer stem‐like cell (CSC) features in various cancers. We previously reported that CD24‐high ovarian cancer cells showed CSC phenotypes, such as tumourigenicity, chemoresistance and stemness‐related gene overexpression. However, although MET contributes to ovarian cancer progression, its roles in ovarian CSCs and its relation with CD24 remain unknown.

Recurrence of ovarian cancer is frequently observed following chemotherapy, the standard treatment for ovarian cancer. One significant factor contributing to cancer recurrence after chemotherapy is the presence of CSCs. Ovarian cancer is recognized as one of the cancer types strongly influenced by CSC activity.^1^ Therefore, targeting CSC properties accompanied by chemotherapy may be necessary for relapse‐free treatment. Presently, the standard approach for treating ovarian cancer involves a combination of debulking surgery and chemotherapy.^2^ Debulking surgery aims to remove cancerous tissue in the abdominal region of ovarian cancer patients. On the other hand, chemotherapy is administered after surgery to eliminate any remaining cancer cells or shrink the tumour size before surgery. Despite implementing optimal surgery and appropriate first‐line chemotherapy, approximately 70%–80% of patients with ovarian cancer experience a recurrence.^3^ The recurrence of cancer is often attributed to drug resistance, which can be caused by various factors, including epigenetic changes, drug inactivation, alterations in drug targets, drug efflux mechanisms, DNA damage repair mechanisms, inhibition of cell death or epithelial‐to‐mesenchymal transition.^4^ In more recent studies, CSCs have emerged as a significant contributor to cancer recurrence.^5^, ^6^

Despite ongoing debates regarding CSCs,^7^, ^8^ there is compelling evidence supporting the existence of ovarian cancer CSCs. The surface epithelial cells of the human ovary possess multipotent capabilities and are considered the source of adenocarcinoma.^9^ Furthermore, a tumourigenic clone of epithelial ovarian cancer cells displayed spheroid growth under in vitro anoikis conditions, a characteristic feature of CSCs.^10^ Several potential markers have been identified for ovarian CSCs, including CD24, CD44, CD117, CD133, EpCAM, ROR1, ALDH, SOX2, OCT4 and NANOG.^6^ Our previous study demonstrated that CD24‐positive ovarian cancer cells exhibited tumourigenicity in a mouse model and showed chemoresistance.^11^ Conversely, Meng et al. revealed that CD44‐positive/CD24‐negative ovarian cancer cells showed stem cell‐like properties and were associated with poor prognosis.^12^

CD24, acting as a signal transducer, is linked to advancing tumours and suppressing the immune system.^13^ It also controls the expression of miRNAs that function as both oncomiRs and tumour suppressors.^14^ However, its role in CSCs remains unclear. Unlike CD24, MET is identified as a marker for pancreatic CSCs, particularly in CD24‐positive CSCs.^15^, ^16^ In triple‐negative breast cancer (TNBC) patients, CD24 and MET markers are associated with poorer outcomes.^17^ MET signalling has been found to induce stem‐like characteristics in glioblastoma and prostate cancer.^18^, ^19^ Additionally, MET plays a role in CSC‐associated traits such as resistance to chemotherapy and anoikis.^20^, ^21^ Considering that CD24 regulates miRNA expression and multiple miRNAs regulate MET,^22^ it is plausible that CD24 induces the expression of MET by controlling miRNA activity, thus conferring stem‐like properties to ovarian cancer.

This study investigated the correlation between CD24 and MET in ovarian cancer and whether CD24 induced MET upregulation via miRNA regulation, which is associated with CSC features.

## 2 MATERIALS AND METHODS

### 2.1 Patient selection and collection of clinical data

Tissue samples from 64 patients with ovarian papillary serous carcinoma were obtained from the archives of the Department of Pathology, Yonsei University College of Medicine. Clinical data were collected from medical records, including patient age, tumour size, stage, preoperative serum level of CA19‐9 and CEA, tumour recurrence and time to recurrence. This study (IRB: 4‐2019‐1301) was approved by the Institutional Review Board, Severance Hospital, Yonsei University, College of Medicine, with a waiver of informed consent.

### 2.2 Immunohistochemical staining

For immunohistochemical analysis, formalin‐fixed, paraffin‐embedded tissue sections were used for validation of immunostaining with anti‐human MET (1:300, Abcam, Cambridge, UK) and anti‐human CD24 (1:100, Santa Cruz Biotechnology, Santa Cruz, CA). Immunohistochemistry (IHC) was performed according to standard IHC techniques. Membranous staining of MET and cytoplasmic staining of CD24 were considered positive and evaluated by light microscopy. Three different scales defined immunohistochemical staining results: (1) ‘Volume’, the proportion of stained cells; (2) ‘Volume × intensity’, staining intensity was classified as 0 (negative), 1 (weak), 2 (moderate) and 3 (strong) and the proportion of stained cells was multiplied by the staining intensity; and 3) ‘The modified score’ was determined as follows: 0, when less than 5% of tumour cells were weakly stained; 1, when 5%–50% of tumour cells were stained with any intensity or less than 5% of tumour cells were moderately or strongly stained; 2, when more than 50% and equal to or less than 90% more than 50% of tumour cells were stained with any intensity; and 3, when more than 90% of tumour cells were stained with any intensity. The modified score was regrouped for statistical analysis into negative (0, 1) and positive (2, 3).

### 2.3 miRNA microarray screening and target gene prediction

Total RNA was isolated from cells using an RNeasy Protect Mini Kit (Qiagen, Hilden, Germany) according to the manufacturer's protocol. The total RNA sample (100 ng) containing miRNA was labelled with Cyanine 3‐pGp (Cy3) using the Agilent miRNA Complete Labeling and Hyb Kit (Agilent Technologies, Santa Clara, CA). The sample was placed on an Agilent Human miRNA v15 and covered by the gasket slide (Agilent Technologies). Slides were hybridized for 20 hr at 55°C using the Agilent hybridization system, washed at room temperature for 5 min each in GE Wash Buffer 1, then GE Wash Buffer 2 (Agilent Technologies, Santa Clara, CA) and then centrifuged at 3000 rpm for 20 s to dry. miRNA arrays were analysed using GeneSpring GX v11 (Agilent Technologies, Santa Clara, CA). Data were applied to standard normalization methods for one‐channel microarrays, background subtraction and percentile median normalization. Fold‐change values were calculated for unpaired comparisons with controls and averaged to generate a mean fold change. Welch's *t*‐test was used to detect significant changes (*p*‐value <0.05). The target prediction of miRNAs was surveyed using TargetRank, miRWalk and TargetScan.

### 2.4 Putative transcription factor binding site identification and chromatin immunoprecipitation

Putative transcription factors (TFs) binding to MIR181A1 and MIR181A2 promoters were analysed in their upstream 4 kb using PROMO^23^ and BDGP.^24^ TF candidates were limited to the TFs existing in BDGP‐predicted regions among the PROMO‐predicted TFs. Chromatin immunoprecipitation (ChIP) assay was performed using Pierce Agarose ChIP Kit (ThermoFisher Scientific, Waltham, MA) according to the manufacturer's instructions. Briefly, cells were fixed with formaldehyde, washed with ice‐cold PBS and centrifuged. After discarding PBS, the cells were lysed and added with 10 U/ul of Micrococcal Nuclease for 15 min at 37°C for chromatin digestion. For immunoprecipitation, anti‐YY1 antibody (Abcam, Cambridge, UK) were added to the supernatants containing digested chromatins and incubated overnight at 4°C on Adjustable‐Angle Rotator (FINEPCR, Gunpo‐si, Korea). ChIP Grade Protein A/G plus Agarose (Abcam, Cambridge, UK) was added to IP reactions and incubated overnight at 4°C with rotation. After incubation, DNAs were eluted using columns and then recovered. The enrichment of binding sites was performed using real‐time PCR. Primers were provided in Table S1.

### 2.5 Patient survival and drug response analyses

The patient survival associated with gene expression and chemotherapy was analysed using KM plotter.^25^ Gene expression difference between non‐responders and responders to chemotherapy was analysed using ROC plotter.^26^

### 2.6 Statistical analysis

Spearman correlation analysis was performed to evaluate the correlation of CD24 and MET expression on immunohistochemistry. These data were analysed using the R for Windows version 2.15.3 (R Foundation for Statistical Computing). Cox regression analysis for factors affecting tumour recurrence and patient survival, the Kaplan–Meier method and the log‐rank test for the survival curve were conducted using SAS (version 9.2). *P* values of less than 0.05 were considered statistically significant. The statistical significance of gene expression in cells and correlation in microarray and NGS datasets was determined by student's *t*‐test (two‐tailed, standard deviation) and Pearson's correlation coefficient using Prism (GraphPad Software, La Jolla, CA). Asterisks were used to indicate p values: one for *p* ≤ 0.05, two for *p* ≤ 0.01 and three for *p* ≤ 0.001.

The materials and methods for cell culture, flow cytometry‐based assays, gene manipulation, luciferase reporter plasmid construction and dual luciferase assays, real‐time PCR analysis, Western blot analysis, half maximal inhibitory concentration (IC50) determination, colony formation assay and graphical presentation were provided in Data S1.

## 3 RESULTS

### 3.1 CD24 and MET expression affected patient survival and sensitivity to platinum‐based chemotherapy in ovarian cancer

**FIGURE 1:**
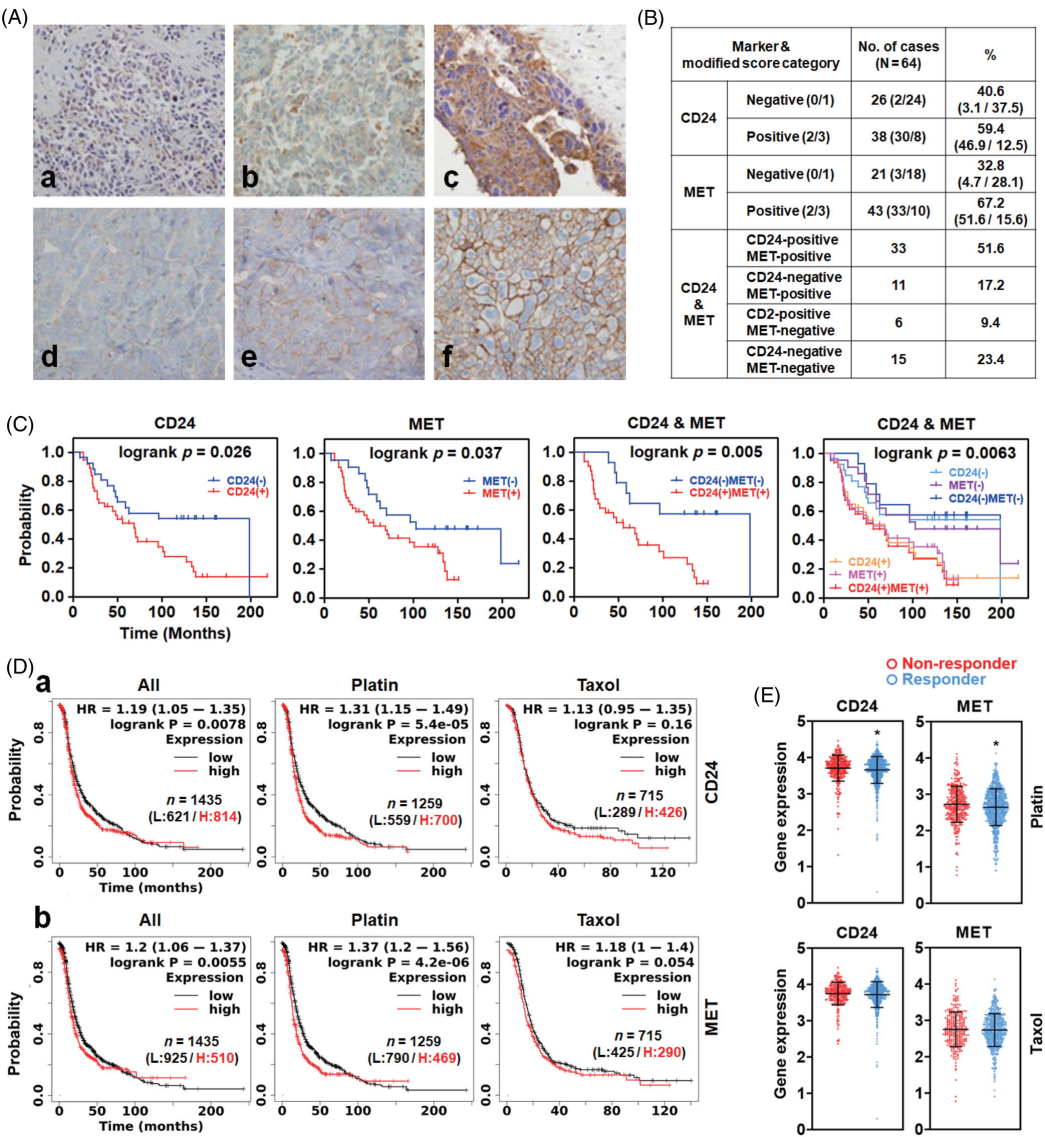
The effect of CD24 and MET expression in poor patient survival and low responses to Platin‐based chemotherapy in ovarian cancer. (A) Representative images of immunohistochemical staining for CD24 and MET in ovarian serous carcinoma. Representative images of expression of CD24 with intensity 1 (a), 2 (b) and 3 (c). Representative images of expression of MET with intensity 1 (d), 2 (e) and 3 (f). (B) Immunohistochemical staining results for CD24 and MET. (C) Patient survival analysis associated with CD24 and MET expression in ovarian cancer. Patient survivor curves were plotted using the immunohistochemistry staining results. (D) Survival analysis of ovarian cancer patient groups with low and high expression of (a) CD24 and (b) MET under chemotherapy. The survival curves were plotted using the KM plotter. E) Expression analysis of CD24 and MET between non‐responder (N) and responder (R) groups to Platin (*N* = 353, *R* = 804) or Taxane (*N* = 269, *R* = 568). The graphs were plotted with the values from ROC plotter using Prism. Asterisks present *p* values: * for *p* ≤ 0.05.

A tissue immunostaining‐based survival analysis examined how CD24 and MET expression affected ovarian cancer patient survival. The clinical characteristics of 74 patients used for the analysis were listed in Table S2. The immunohistochemical staining for CD24 and MET was classified into intensities 1–3 (Figure 1A). In Figure 1B, of the 64 ovarian serous papillary carcinoma cases, 38 (59.4%) and 43 (67.2%) were positive for CD24 and MET immunostaining, respectively. In more than 70% of cases, tumour cells showed modified scores of 1 or 2 for CD24 and MET expression, indicating their inhomogeneous expression. 33/11/6/15 cases were CD24+MET+/CD24‐MET+/CD24+MET‐/CD24‐MET‐, respectively. The tumour cells at the tip of the papillae and stromal invasion were more intensely and frequently stained for CD24 and MET (Figure S1). In Table S3, none of the clinical parameters was associated with disease‐free survival (DFS). Higher CD24 expressions measured by three different scales, that are CD24 volume, volume × intensity and the modified score, were all significantly associated with shorter DFS (*p* = 0.0119, 0.0048 and 0.0387) and the hazard ratio of positive cases for CD24 relative to negative cases was 3.558. Similarly, higher MET expression measured by the same three scales showed a significant correlation with shorter DFS (*p* = 0.0062, 0.0057 and 0.0459), and the hazard ratio of positive cases for MET relative to negative cases was 2.951. In addition, immunopositivity for both CD24 and MET correlated with a shorter DFS, and the hazard ratio was 7.705 (*p* = 0.0464). Regarding overall survival, no factors showed a significant correlation (data not shown). On multivariate analysis, there were no significant factors related to tumour recurrence. In Figure 1C, CD24 immunopositivity correlated with a shorter DFS (*p* = 0.0256), and MET immunopositivity tended to show a decreased DFS, although not statistically significant (*p* = 0.0511). CD24(+)MET(+) significantly correlated with a shorter DFS when compared only to CD24(−)MET(−) patients (*p* = 0.0176). The difference in DFS between CD24(−)MET(−) and CD24(+)MET(+) patients was also evaluated. Although there was no statistical significance but a tendency to differ in a comparison of the DFS of CD24(−)MET(−) versus CD24(−)MET(+)/CD24(+)MET(−) versus CD24(+)MET(+) (*P* = 0.0516). Patient survival was analysed using a KM plotter to confirm whether CD24 and MET expression are related to treatment resistance. In Figure 1D‐a, CD24 expression was significantly associated with a shorter survival probability in a group including all patients and the sub‐group treated with Platin, while it was not in the sub‐group treated with Taxol. Similarly, in Figure 1D‐b, MET expression was significantly associated with a shorter survival probability in a group including all patients and the sub‐group treated with Platin, while it was not in the sub‐group treated with Taxol. The expression level of CD24 and MET was compared between non‐responder and responder groups to Platin and Taxane using the ROC plotter. The responder group to Platin, not Taxane, showed a significantly lower expression of CD24 and MET than the non‐responder group (Figure 1E).

### 3.2 CD24 expression induced the differential expression of miRNAs in ovarian cancer cells

**FIGURE 2:**
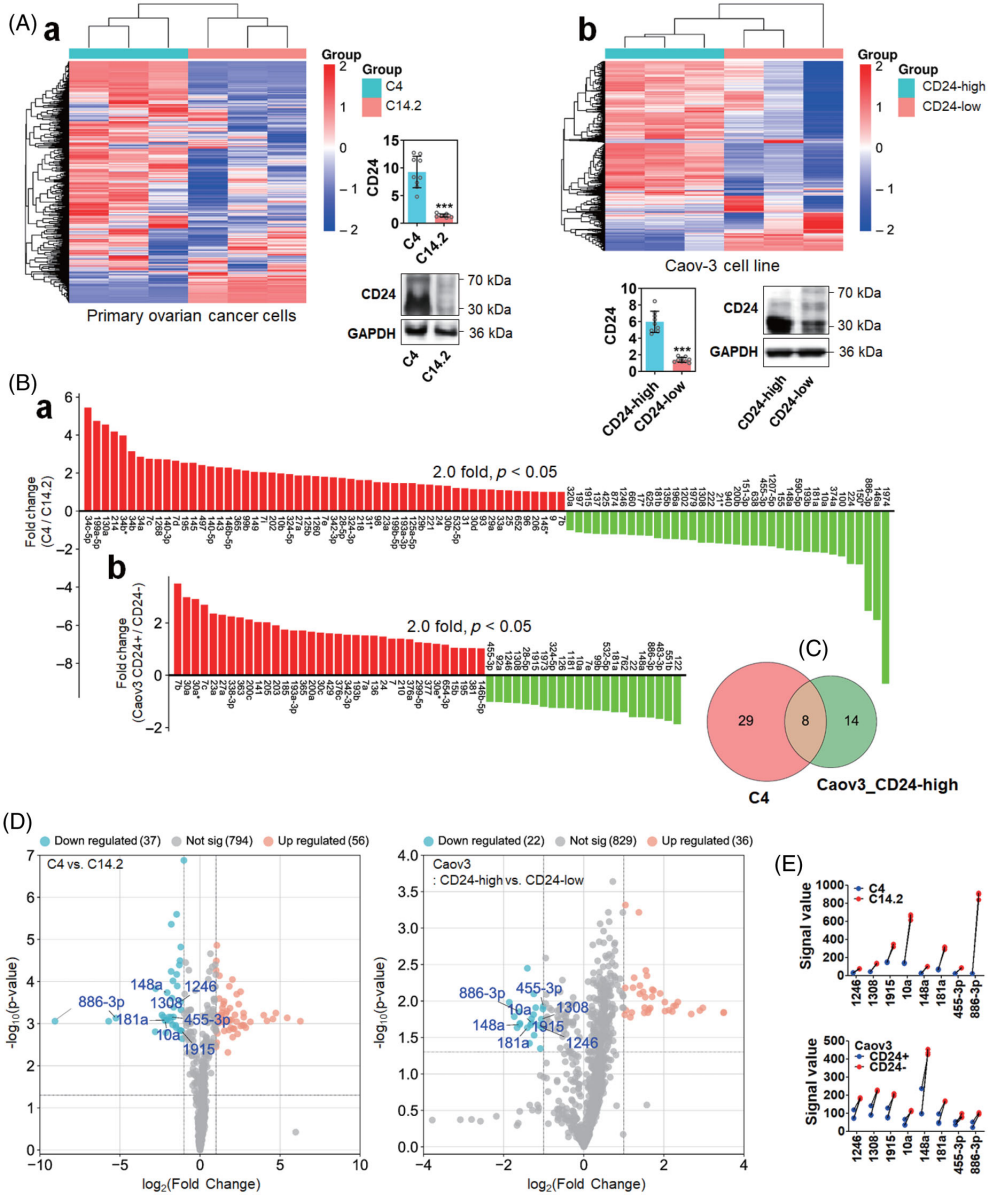
CD24‐dependent expression of miRNAs in ovarian cancer cells. (A) Heatmap presentation of miRNA expression between CD24‐low and CD24‐high populations of (a) primary ovarian cancer cell clones and (b) Caov‐3 cells. Primary cancer cell clones were derived from an ovarian cancer patient. CD24 expression in cell clones and populations was confirmed using real‐time PCR and Western blot analyses. Asterisks present p values: *** for *p* ≤ 0.001. (B) Fold change of miRNA expression between CD24‐low and ‐high subgroups of primary ovarian cancer cells and Caov‐3 cells. Fold changes were determined by calculating the increase or decrease of the CD24‐high population relative to the CD24‐low population. (C) Venn diagram presentation of the miRNAs commonly downregulated in C4 and the CD24‐high population of Caov‐3 cells. Presentation of the miRNAs commonly downregulated in the CD24‐high populations of primary ovarian cancer cells and Caov‐3 cells using (D) scatter plot and (E) superimposed symbols with connecting line plot.

CD24 can regulate different signalling pathways^27^ and miRNA expression.^14^ Therefore, we hypothesized that CD24 upregulates MET expression by regulating miRNA expression in ovarian cancer cells. To validate and test our hypothesis, we analysed the CD24‐associated expression of miRNAs in primary ovarian cancer cells and the Caov‐3 cell line. A differential expression pattern of miRNAs was observed between CD24‐low clone (C14.2) and CD24‐high clone (C4) derived from an ovarian cancer patient (Figure 2A‐a), as well as between the CD24‐low and ‐high populations sorted from Caov‐3 cells (Figure 2A‐b). Compared to the C14.2, 56 miRNAs were significantly over 2‐fold upregulated in the C4, while 37 miRNAs were downregulated (Figure 2B‐a). Compared to the CD24‐low population of Caov‐3 cells, 56 miRNAs were significantly over 2‐fold upregulated in the CD24‐high population, while 37 miRNAs were downregulated (Figure 2B‐b). The downregulation of a specific miRNA can be associated with MET upregulation. Therefore, we investigated the miRNAs commonly downregulated in the C4 and the CD24‐high population of Caov‐3 and found 8 miRNAs (Figure 2C). The fold changes and *p* values of the downregulated miRNAs (miR‐10a, 148a, 181a, 455‐3p, 886‐3p, 1246, 1308 and 1915) were distributed as shown in Figure 2D. Among the 8 miRNAs, miR‐886‐3p showed the largest differential expression between the C14.2 and C4, while miR‐148a showed the largest differential expression between the CD24‐low and ‐high populations of the Caov‐3 cells (Figure 2E). The miRNAs commonly upregulated in C4 and the CD24‐high population of Caov‐3 cells, their distribution with fold changes and *p* values and differential expression were provided in Figure S2.

### 3.3 MET overexpression was associated with miR‐181a downregulation in CD24‐high ovarian cancer cells

**FIGURE 3:**
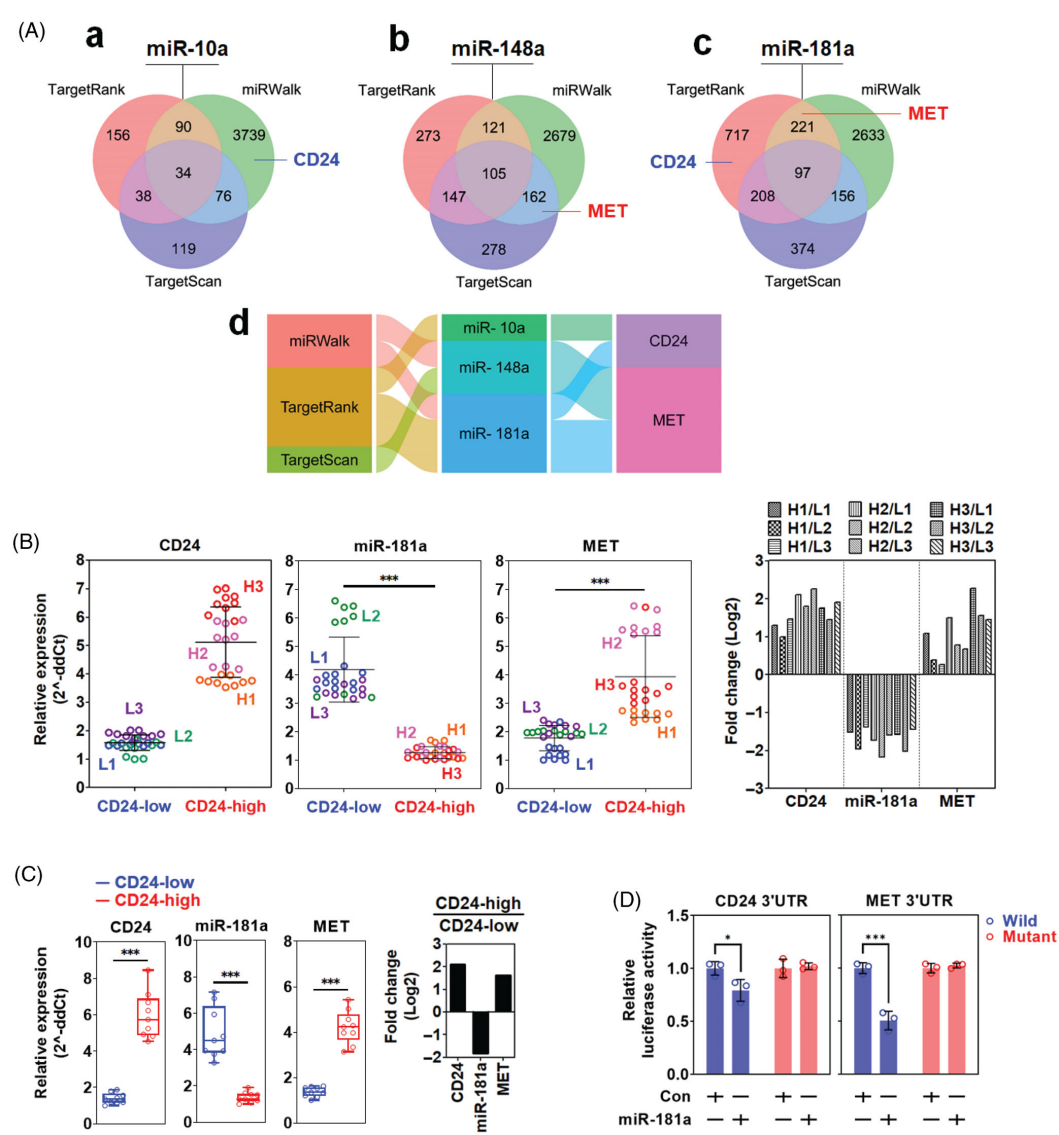
Correlation between CD24, miR‐181a and MET expression in ovarian cancer. (A) Venn diagram presentation of putative target gene analysis of (a) miR‐10a, (b) miR‐148a and (c) miR‐181a. (d) Summary of putative target gene analysis using Alluvial plot. (B) Real‐time PCR analysis of MET and miR‐181a expression in primary ovarian cancer cell clones with CD24‐low and CD24‐high expression. L1, L2 and L3 were CD24‐low clones. H1, H2 and H3 were CD24‐high clones. The fold changes of CD24, miR‐181a and MET were presented by each combination of CD24‐high clone to CD24‐low clone. (C) Real‐time PCR analysis of MET and miR‐181a expression in the CD24‐low and ‐high populations of Caov‐3 cells. (D) Binding analysis of miR‐181a to the 3'UTR of CD24 and MET. Asterisks present p values: * for *p* ≤ 0.05 and *** for *p* ≤ 0.001.

The putative target genes of miR‐10a, 148a, 181a and 886‐5p, which were the top 4 downregulated miRNAs, were analysed to validate whether they can target CD24 and MET using 3 miRNA databases (Target Rank, miRWalk and TargetScan). In the analysis, miR‐10a was not predicted to bind to MET on all databases but to bind to CD24 on miRWalk (Figure 3A‐a). miR‐148a was predicted to bind to MET on miRWalk and TargetScan (Figure 3A‐b). miR‐181a was predicted to bind to MET on miRWalk and TargetScan, whereas CD24 on TargetRank (Figure 3A‐c). miR‐886‐5p was absent on those databases. All prediction was summarized in Figure 3A‐d. Since we were interested in the relationship between CD24 and MET, miR‐181a was chosen to proceed with our study. To examine whether miR‐181 downregulation was accompanied by MET upregulation in ovarian cancer cells with high CD24 expression, the other 6 clones derived from the ovarian cancer patient (3 clones for CD24‐low and 3 for CD24‐high) were additionally analysed. In Figure 3B, the expression of miR‐181a was significantly downregulated in three clones with high CD24 expression compared to those with low CD24 expression, while that of miR‐181a was significantly upregulated. The expression fold changes of CD24, miR‐181a and MET in each combination of CD24‐high clone to CD24‐low clone showed a similar trend. In the analysis with the Caov‐3 cells, miR‐181a expression was significantly downregulated in the CD24‐high population compared to the CD24‐low population, while MET expression was significantly upregulated. The expression fold changes of CD24, MET and miR‐181a in the CD24‐high population to the CD24‐low population showed a similar trend to those of primary ovarian cancer cell clones (Figure 3C). The reverse correlation of miR‐181a with CD24 and MET led us to examine their binding. In Figure 3D, the luciferase activity of wild‐type CD24 3'UTR was significantly decreased by transfection with miR‐181a, while that of mutant CD24 3'UTR was not. Like CD24, the luciferase activity of wild‐type MET 3'UTR was significantly reduced by transfection with miR‐181a, while that of mutant MET 3'UTR was not. The wild and mutant plasmid constructs of CD24 and MET 3′ UTR were provided in Figure S3.

### 3.4 miR‐181a was negatively correlated in expression with CD24, MET and stemness‐related genes, while positively correlated with chemosensitivity and patient survival

**FIGURE 4:**
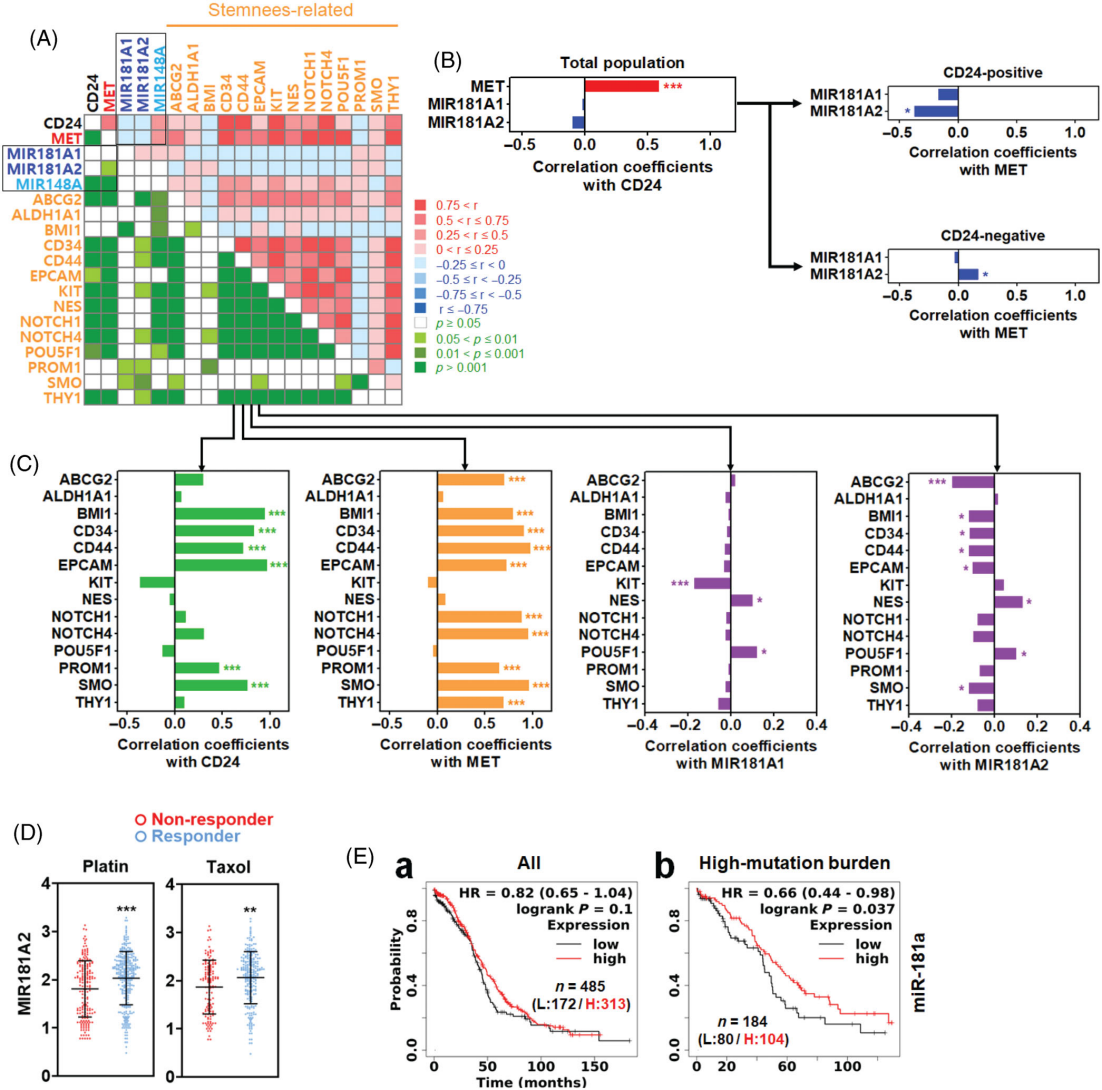
Expression correlation analysis between CD24, MET, miR‐181a and stem cell‐related genes in ovarian cancer. (A) Matrix graph presentation of correlation between CD24, MET, miR‐181a and stem cell‐related genes. Bar graph presentation of (B) correlation between CD24, MET and miR‐181a and (C) correlation between CD24, MET, miR‐181a and stemness‐related genes. (D) Expression analysis of miR‐181a between non‐responder (N) and responder (R) groups to Platin (*N* = 187, *R* = 303) or Taxane (*N* = 137, *R* = 235). The graphs were plotted with the values from ROC plotter using Prism. (E) Survival analysis of ovarian cancer patient groups (a: all, b: high mutation burden) with low and high expression of miR‐181a. The survival curves were plotted using the KM plotter. Asterisks present *p* values: * for *p* ≤ 0.05, ** for *p* ≤ 0.01 and *** for *p* ≤ 0.001.

The reverse correlation of miR‐181a with CD24, MET and stemness‐related genes was further evaluated using the TCGA‐OV dataset. Some stemness‐related genes were positively correlated with CD24.^11^ In Figure 4A, CD24 was positively and significantly correlated in expression with MET and most stemness‐related genes while negatively but not significantly associated with MIR181A1 and MIR181A2 (precursors for mature miR‐181a). MET was positively and significantly correlated in expression with stemness‐related genes but negatively and significantly with MIR181A2. CD24 and MET were positively and significantly correlated with MIR148A. Since CD24 was not significantly and negatively correlated with MIR181A1 or MIR181A2 in the total population, the correlation of CD24 with MIR181A1 and MIR181A2 was further analysed in CD24‐positive and ‐negative populations. As shown in Figure 4B, CD24 was significantly and negatively correlated in expression with MIR181A2, while it was significantly and positively correlated with MIR181A2 in the CD24‐negative population. In the correlation analysis with stemness‐related genes, CD24 was significantly and positively correlated with BMI, CD34, CD44, EPCAM, PROM1 and SMO. MET was significantly and positively correlated with ABCG2, BMI1, CD34, CD44, EPCAM, NOTCH1, NOTCH2, PROM1, SMO and THY1. MIR181A1 was significantly and negatively correlated with KIT but positively with NES and POU5F1. MIR181A2 was significantly and negatively correlated with ABCG2, BMI1, CD34, CD44, EPCAM and SMO, whereas positively with NES and POU5F1 (Figure 4D). The correlation analysis led us to assume that MIR181A2 is related to chemosensitivity, a phenotype of CSCs. Therefore, the relationship between MIR181A2 expression and chemosensitivity was analysed using the ROC plotter. Responders to Platin and Taxane‐based treatment showed a significantly higher expression of MIR181A2 than non‐responders (Figure 4D). In the patient survival analysis with KM plotter, the high expression level of miR‐181a was not significantly associated with more prolonged survival than the low expression level in all patient populations (Figure 4E‐a). On the other hand, the high expression level of miR‐181a was significantly associated with more prolonged survival than the low expression level in the patient population with high‐mutant burden (Figure 4E‐b).

### 3.5 CD24 regulated MET expression via YY1‐dependent miR‐181a downregulation

**FIGURE 5:**
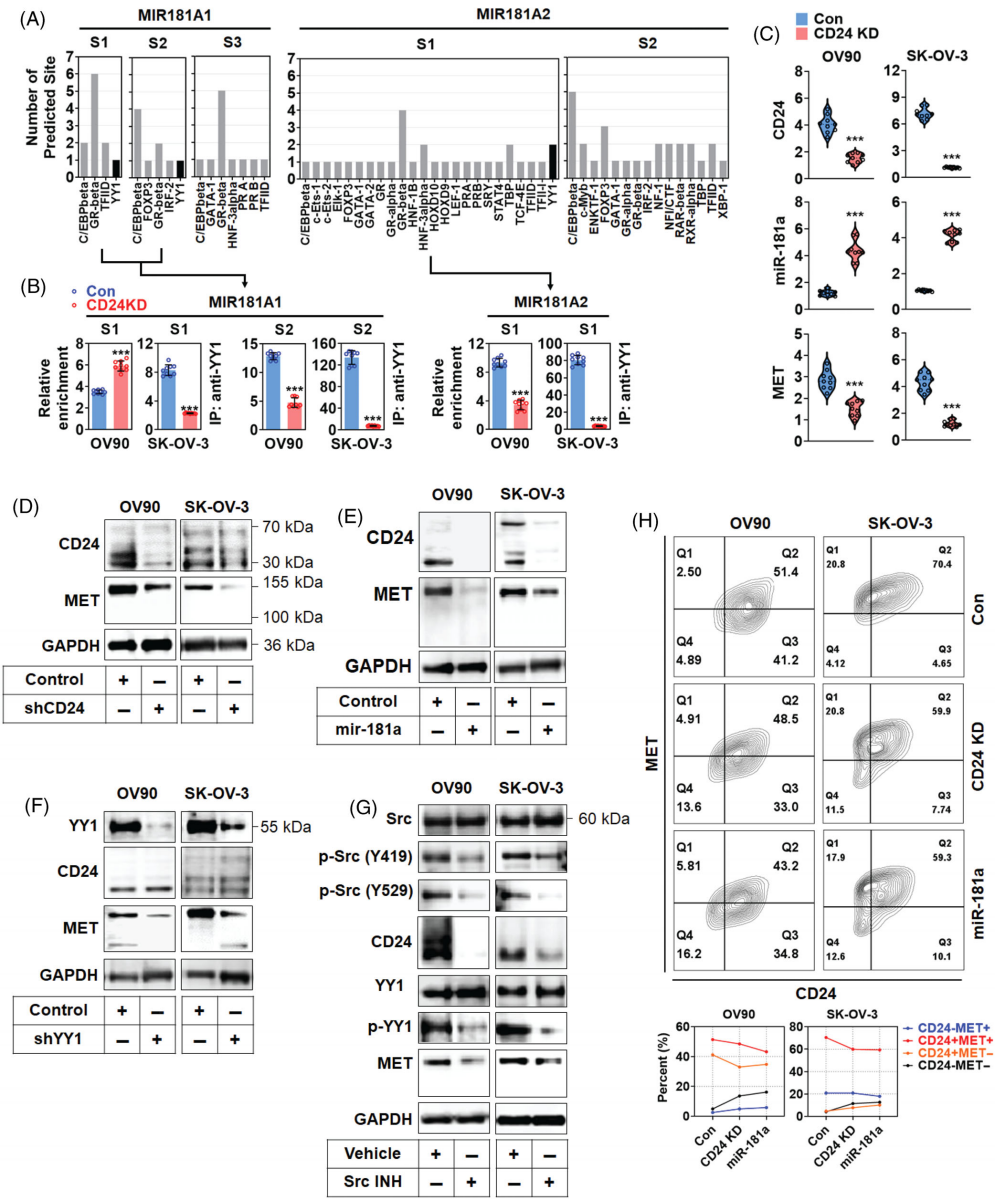
CD24‐dependent miR‐181a downregulation by Src‐associated YY1 activation. (A) Transcription factor binding site analysis of miR‐181a promoter. The upstream 4 kb sequence of MIR181A1 and MIR181A2 were analysed using PROMO and BDGP. (B) Chromatin immunoprecipitation analysis. Relative enrichment was validated using real‐time PCR analysis. (C) Real‐time PCR analysis of miR‐181a and MET expression in CD24‐knockdown ovarian cancer cells. Western blot analysis of (D) CD24‐dependent MET expression, (E) CD24 and MET expression in miR‐181a‐overexpressing ovarian cancer cell lines, (F) CD24 and MET expression in YY1‐knockdown ovarian cancer cell lines and (G) the expression of CD24, YY1 and MET, and the phosphorylation of YY1 in the ovarian cancer cells treated with Src inhibitor. (H) Flow cytometry analysis of CD24 and MET expression in CD24‐knockdown or miR‐181a‐overexpressing ovarian cancer cells. Asterisks present p values: *** for *p* ≤ 0.001.

The promoter upstream 4 kb of MIR181A1 and MIR181A2 was analysed to find putative transcription factors binding to them. As shown in Figure 5A, many transcription factors potentially bind to the promoter regions of MIR181A1 and MIR181A2. Among them, YY1 was the transcription factor that commonly binds to MIR181A1 and MIR181A2. There were three sites for YY1 in MIR181A1 and two for YY1 in MIR181A2. To confirm the binding of YY1 to the putative sites, chromatin immunoprecipitation with anti‐YY1 antibody was performed using the OV90 and SK‐OV‐3, which showed the high expression of CD24, transfected with control or shCD24 plasmid. In Figure 5B, the sequence enrichment of the site‐1 (S1) on the MIR181A1 promoter was significantly reduced in SK‐OV‐3 cells by CD24 knockdown but not in OV90 cells. The sequence enrichment of the site (S2) on the MIR181A1 promoter was significantly decreased in OV90 and SK‐OV‐3 cells by CD24 knockdown. The sequence enrichment of site (S1) on the MIR181A2 promoter was significantly reduced in OV90 and SK‐OV‐3 cells by CD24 knockdown. In real‐time PCR analysis, CD24 knockdown induced the upregulation of miR‐181a transcript and the downregulation of MET transcript in OV‐90 and SK‐OV‐3 cells (Figure 5C). Similar to transcript analysis, CD24 knockdown decreased the expression of MET protein in OV90 and SK‐OV‐3 cells (Figure 5D) and miR‐181a overexpression reduced the expression of CD24 and MET proteins (Figure 5E). YY1 knockdown also decreased the expression of CD24 and MET proteins in OV90 and SK‐OV‐3 cells (Figure 5F), whereas it increased the expression of miR‐181a (Figure S4). According to previous studies, Src can be activated by CD24^14^ and Src can induce YY1 phosphorylation.^28^ Therefore, the phosphorylation of Src and YY1 and expression of CD24 and MET proteins were analysed using a Src inhibitor. As shown in Figure 5G, the phosphorylation of YY1 (S365) and Src (Y419 and Y529) and the expression of CD24 and MET proteins was decreased in OV90 and SK‐OV‐3 cells by treatment with the Src inhibitor. In flow cytometry analyses, CD24 knockdown mainly decreased the populations of CD24+MET+ and CD24+MET− in OV90 and SK‐OV‐3 cells (Figure 5H).

### 3.6 CD24, miR‐181a and MET expression were associated with cancer stem‐like phenotypes in ovarian cancer

**FIGURE 6:**
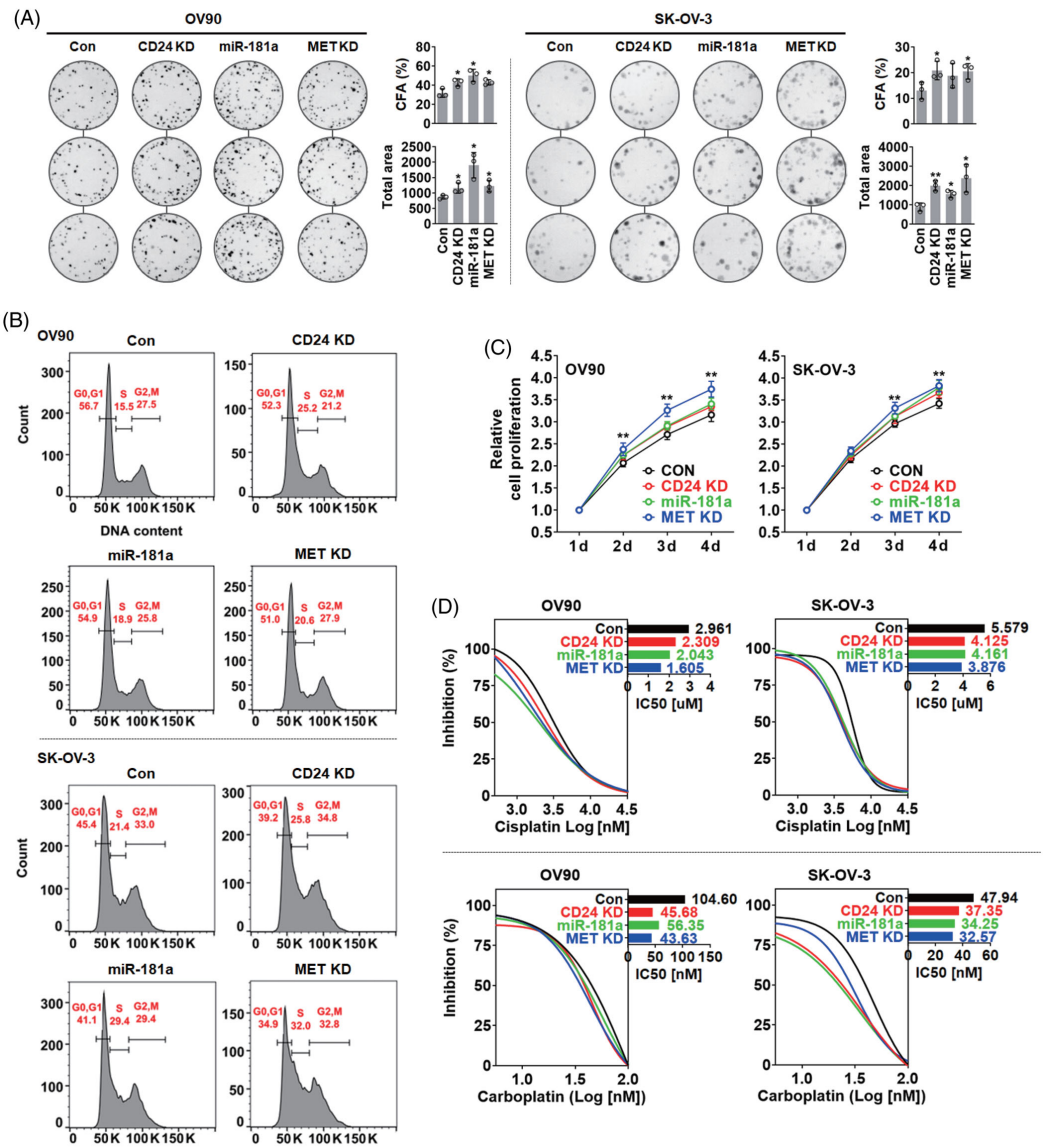
Cancer stem‐like cell phenotype manifestation by CD24, miR‐181a and MET expression. Comparative analyses of (A) colony formation, (B) cell cycle, (C) proliferation and (D) half‐maximal inhibitory concentration between CD24‐knockdown, miR‐181a‐overexpressing and MET‐knockdown ovarian cancer cells. CFU: Colony forming ability. Asterisks present *p* values: * for *p* ≤ 0.05 and ** for *p* ≤ 0.01.

Our previous study demonstrated that CD24 expression was associated with the CSC phenotypes of ovarian cancer cells, such as tumourigenesis, chemoresistance and stem‐related gene overexpression.^11^ Therefore, the relationship of CSC phenotype manifestation with CD24, miR‐181a and MET expression was analysed using the assays for colony formation, cell cycle, cell proliferation and chemosensitivity. In colony formation analysis, the colony forming ability (CFU, %) and area of colonies were significantly increased in OV90 and SK‐OV‐3 cells by CD24 knockdown, miR‐181a overexpression and MET knockdown compared to the control (Figure 6A). In cell cycle analysis, the population of S phase was increased in OV90 and SK‐OV‐3 cells by CD24 knockdown, miR‐181a overexpression and MET knockdown. On the other hand, the population of G0/G1 phase was decreased compared to the control (Figure 6B). In cell proliferation analysis, the proliferation of OV90 and SK‐OV‐3 cells was increased by CD24 knockdown, miR‐181a overexpression and MET knockdown compared to control (Figure 6C). The half maximal inhibitory concentration (IC50) of Cisplatin and Carboplatin was decreased in OV90 and SK‐OV‐3 cells by CD24 knockdown, miR‐181a overexpression and MET knockdown compared to control (Figure 6D).

## 4 DISCUSSION

**FIGURE 7:**
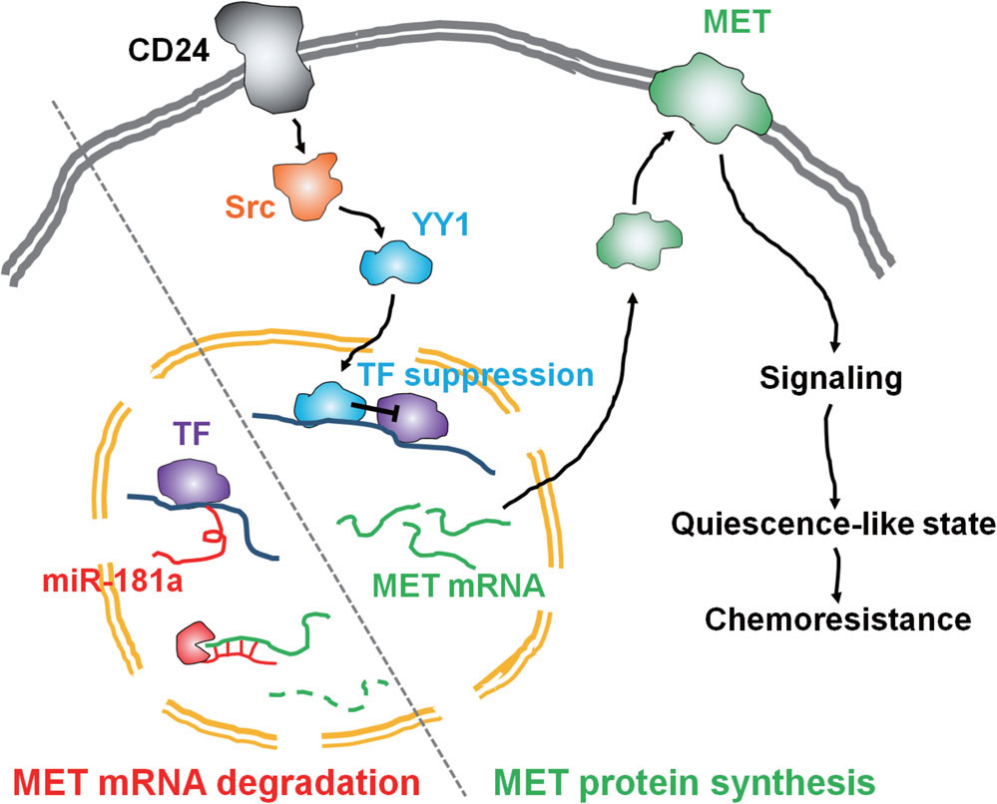
CD24‐mediated signalling route for ovarian cancer stem‐like phenotype manifestation. In the absence of CD24‐mediate signalling, MET mRNA is degraded by miR‐181a. On the other hand, in the presence of CD24‐mediate signalling, the YY1 activated by Src suppresses the transcription of miR‐181a, which leads to MET expression. MET may induce the cellular quiescence‐like state and its following chemoresistance in ovarian cancer cells.

In this study, we showed the co‐expression of CD24 and MET was associated with poorer patient survival than the expression of CD24 or MET alone. Also, we demonstrated CD24 upregulated MET expression via the Src‐mediated downregulation of miR‐181a, which was associated with CSC phenotypes such as cellular quiescence‐like state and chemoresistance (Figure 7).

The roles of CD24 and MET in ovarian CSCs have not been reported. Accumulated studies showed that the expression of CD24 or MET was associated with chemoresistance^11^, ^29^ and recurrence^30^, ^31^ in ovarian cancer. Considering chemoresistance and recurrence are referred to as CSC phenotypes, CD24 and MET are likely to be correlated in ovarian CSCs. In this study, we first hypothesized that CD24 induces MET expression, contributing to the manifestation of CSC phenotypes in ovarian cancer. The opposite direction, CD24 expression by MET, may also be possible since MET is linked to various downstream networks, and its knockdown can induce the upregulation of miR‐181a in OV90 and SK‐OV‐3 cells (Figure S5) Nevertheless, we prioritized CD24 to MET since the relationship between CD24 expression and ovarian cancer was more reported than MET. To mine a downstream effector of CD24 to regulate MET expression, we analysed miRNA transcriptome and found miR‐181a. Except for post‐transcriptional regulation by miRNAs, MET expression can be conventionally regulated by transcription factors. The main reasons why we focused on miRNAs were our interest in the roles of miRNAs in CSCs and the exosomal delivery of miRNAs for CSC therapeutics.

According to our results, CD24 expression induced the downregulation of miR‐181a, contributing to MET overexpression in serous types of ovarian cancer cell lines, OV90 and SK‐OV‐3 cells. This was supported by the expressional correlation between CD24, miR‐181a and MET in TCGA‐OV dataset analysis. And CD24‐induced expressional alteration of miR‐181a and MET was associated with cellular quiescence‐like state and resistance to platinum‐based chemotherapy. However, different findings about the expression and functions of miR‐181a in ovarian cancer were previously reported. Li Zuo et al. found that miR‐181a was upregulated in ovarian cancer patient tissues and circulating tumour cells.^32^ Aditya Parikh et al. demonstrated that miR‐181a promoted TGF‐β‐mediated EMT, and its overexpression increased survival, migration, invasion and drug (cisplatin) resistance in A2780 cells,^33^ histologically unidentified ovarian cancer cell line but putatively endometrioid‐clear cell.^34^

The roles of MET in ovarian CSC phenotype manifestation have not been studied. According to previous studies, MET signalling is known to be essential for CSC maintenance in several cancers, such as colorectal,^35^ breast,^36^ prostate^37^ and glioblastoma.^38^ In ovarian cancer, MET expression was associated with proliferation and lymph node metastasis. Inhibition of MET significantly decreased the proliferation of ovarian clear cell carcinoma (OCCC) cell line RMG1 and increased apoptosis. Also, MET inhibition significantly reduced the tumour weight in a xenograft model with RMG1 cells and a PDX model with OCCC compared to controls.^39^ Meta‐analysis with 568 ovarian cancer patients showed that a high expression level of MET was associated with higher stages and lymph node metastasis rates than low expression.^40^ Considering that CSCs have a part in metastasis,^41^ these previous studies may implicate the relationship between MET and ovarian CSC phenotype manifestation. On the other hand, our study first showed that MET overexpression was associated with CSC‐related phenotypes (cellular quiescence‐like state and chemoresistance) in OV90 and SK‐OV‐3 cells.

This study demonstrated that CD24 upregulated MET expression via the Src‐mediated downregulation of miR‐181a in serous types of ovarian cancer cells, which contributed to cellular quiescence‐like state and chemoresistance. Therefore, the CD24‐miR‐181a‐MET may be a signalling route for ovarian CSC phenotype manifestation, which can be used as potential markers and therapeutic targets for ovarian CSCs.

## AUTHOR CONTRIBUTIONS

All authors of this article have read and approved the final version submitted and have directly participated in the planning, execution, analysis or writing of the study. Ji Eun Kwon performed immunochemical staining, analysed clinical data, arranged the data, performed statistical analysis and drafted the manuscript. Yeonsue Jang drafted the manuscript, raised the funding and mainly conducted in vitro works. Bo Seong Yun and Yon Hee Kim collected patient tissues and assisted with immunohistochemistry staining. Suki Kang performed the miRNA microarray. Baek Gil Kim conceived this study, analysed and interpreted the data, raised the funding, supervised this work and wrote the final version of the manuscript. Nam Hoon Cho interpreted the data, supervised this work and wrote the final version of the manuscript.

## FUNDING INFORMATION

This study was supported by the Ministry of Education of the Republic of Korea and the National Research Foundation of Korea (2022R1A2C1006398, Baek Gil Kim; 2021R1I1A1A01042938, Yeonsue Jang).

## CONFLICT OF INTEREST STATEMENT

We declare there are no conflicts of interest associated with this work.

## Supporting information


**Figure S1.** The expression patterns of CD24 and MET in ovarian cancer patient tissues.
(A) Representative cases of CD24 expression assessed by the modified score 0 (a), 1 (b), 2 (c) and 3 (d). (B) Intense staining of CD24 in the tumour cells located at the papillary tip. (C) A frequent pattern of CD24 expression in the tumour cells invading stroma. (D) Representative images of MET expression. (a) Diffuse staining of MET (the modified score 3), (b) MET staining in the tumour cells at the papillary tip, (c) A frequent pattern of MET expression in the tumour cells invading stroma.

**Figure S2.** CD24‐upregulated expression of miRNAs in ovarian cancer cells.
(A) Venn diagram presentation of the miRNAs commonly upregulated in C4 and the CD24‐high population of Caov‐3 cells. Presentation of the miRNAs commonly upregulated in the CD24‐high populations of primary ovarian cancer cells and Caov‐3 cells using (B) Scatter plot and (C) Superimposed symbols with connecting line plot.

**Figure S3.** Plasmid constructs for binding assay.
The wild and mutant plasmid constructs of (A) CD24 and (B) MET 3'UTR.

**Figure S4.** Alteration of miR‐181a expression in ovarian cancer cells by YY1 knockdown.
miR‐181a expression was analysed in the OV90 and SK‐OV‐3 cells transfected with control or YY1 shRNA using real‐time PCR.

**Figure S5.** Alteration of miR‐181a expression in ovarian cancer cells by MET knockdown.
miR‐181a expression was analysed in the OV90 and SK‐OV‐3 cells transfected with control or MET shRNA using real‐time PCR.

**Table S1.** Primers for gene expression analysis and ChIP enrichment analysis.

**Table S2.** Clinical characteristics of the 64 patients with ovarian serous papillary carcinoma.

**Table S3.** Cox regression analysis for factors affecting disease‐free survival.

**Data S1.** Supporting information.

## Data Availability

The raw and processed data of the microarray are available in the GEO database (GSE164748).
