# Supplementary figures and images for: MET overexpression in ovarian cancer via CD24‐induced downregulation of miR‐181a: A signalling for cellular quiescence‐like state and chemoresistance in ovarian CSCs
Source: Cell Prolif. 2023 Nov 29;57(5):e13582. doi: 10.1111/cpr.13582 (PMC11056702; doi:10.1111/cpr.13582)

**Supplementary Figure S1.**

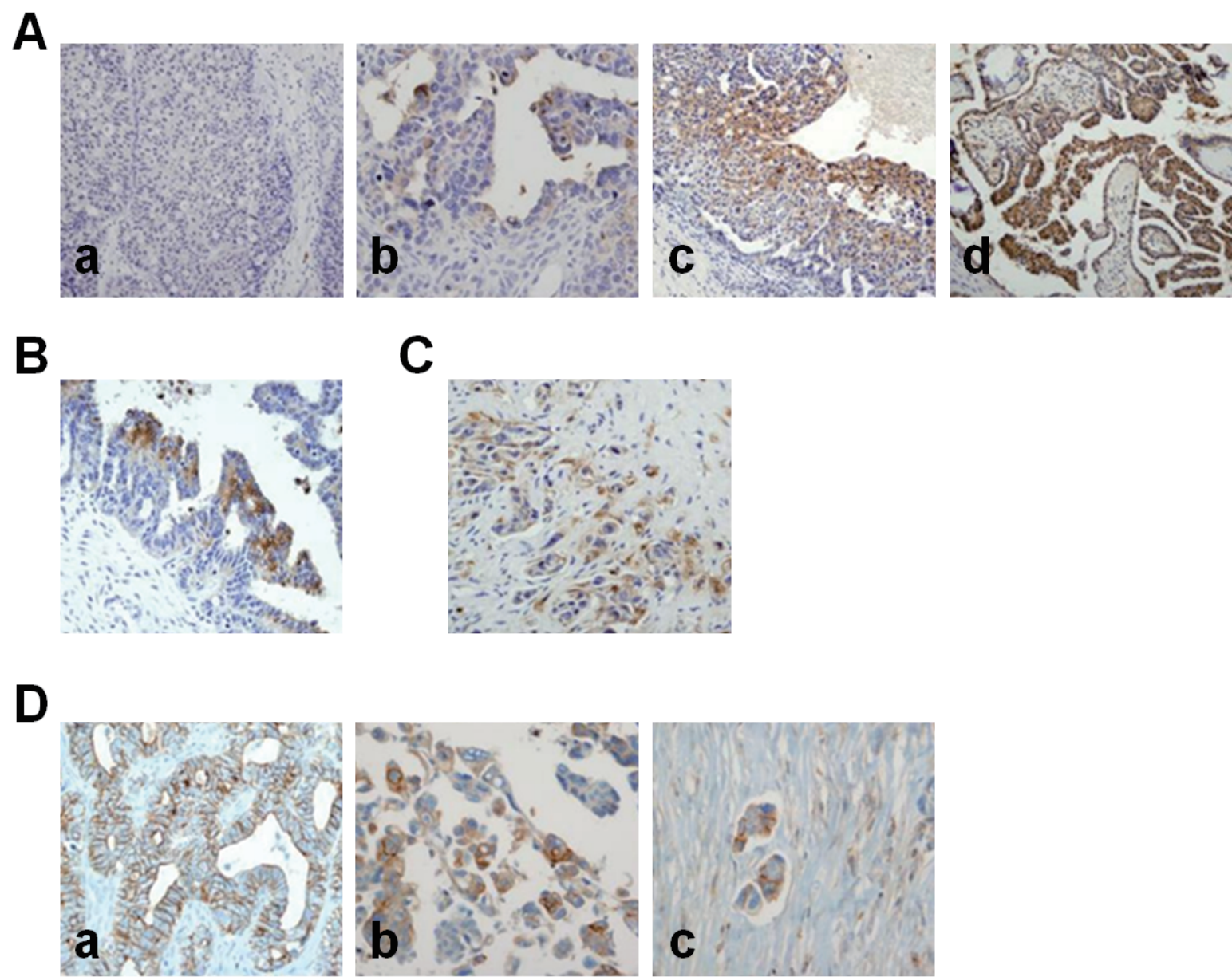

Supplement: Supplementary file 1 — Figure S1. The expression patterns of CD24 and MET in ovarian cancer patient tissues. (A) Representative cases of CD24 expression assessed by the modified score 0 (a), 1 (b), 2 (c) and 3 (d). (B) Intense staining of CD24 in the tumour cells located at the papillary tip. (C) A frequent pattern of CD24 expression in the tumour cells invading stroma. (D) Representative images of MET expression. (a) Diffuse staining of MET (the modified score 3), (b) MET staining in the tumour cells at the papillary tip, (c) A frequent pattern of MET expression in the tumour cells invading stroma. [file CPR-57-e13582-s008.pdf]

# Supplementary Figure S2.

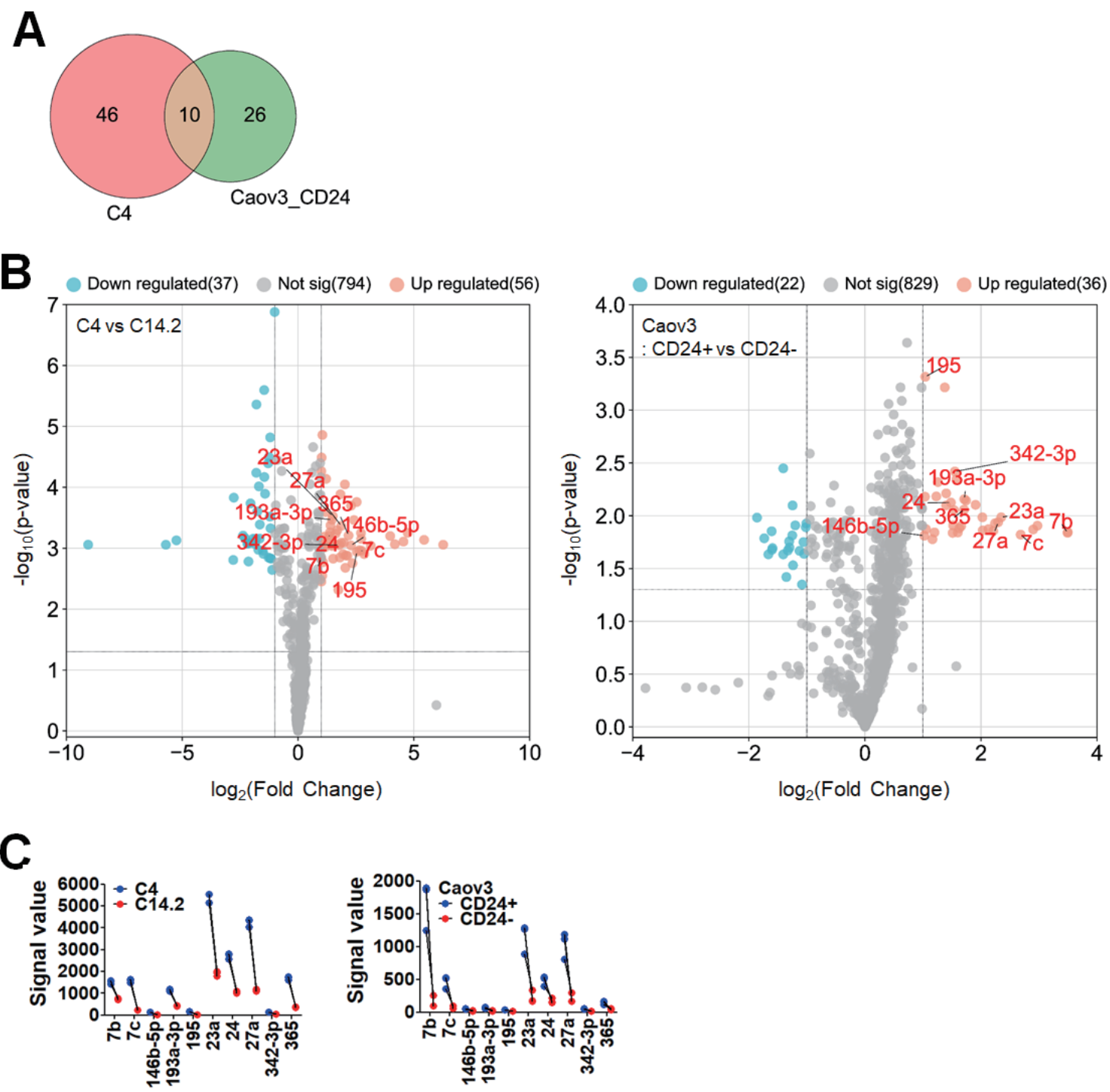

Supplement: Supplementary file 2 — Figure S2. CD24‐upregulated expression of miRNAs in ovarian cancer cells. (A) Venn diagram presentation of the miRNAs commonly upregulated in C4 and the CD24‐high population of Caov‐3 cells. Presentation of the miRNAs commonly upregulated in the CD24‐high populations of primary ovarian cancer cells and Caov‐3 cells using (B) Scatter plot and (C) Superimposed symbols with connecting line plot. [file CPR-57-e13582-s004.pdf]

# Supplementary Figure S3.

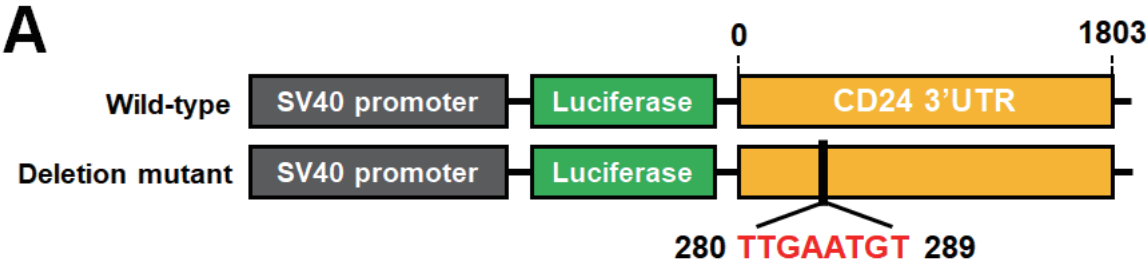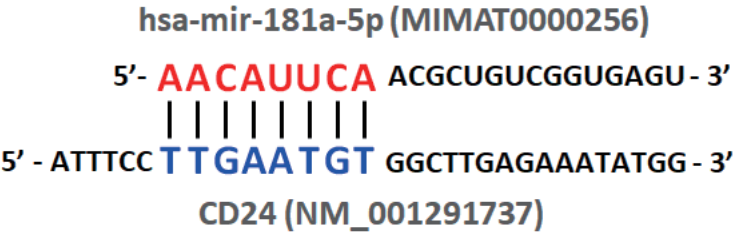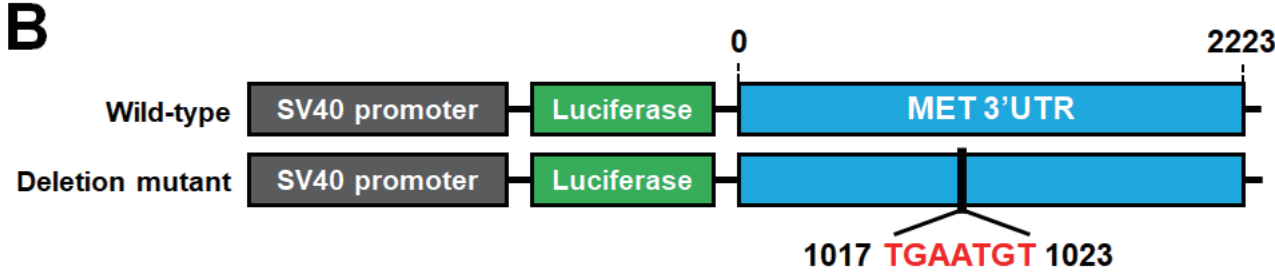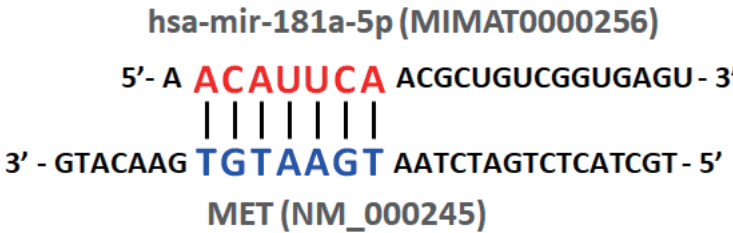

Supplement: Supplementary file 3 — Figure S3. Plasmid constructs for binding assay. The wild and mutant plasmid constructs of (A) CD24 and (B) MET 3'UTR. [file CPR-57-e13582-s003.pdf]

Supplementary Figure S4.

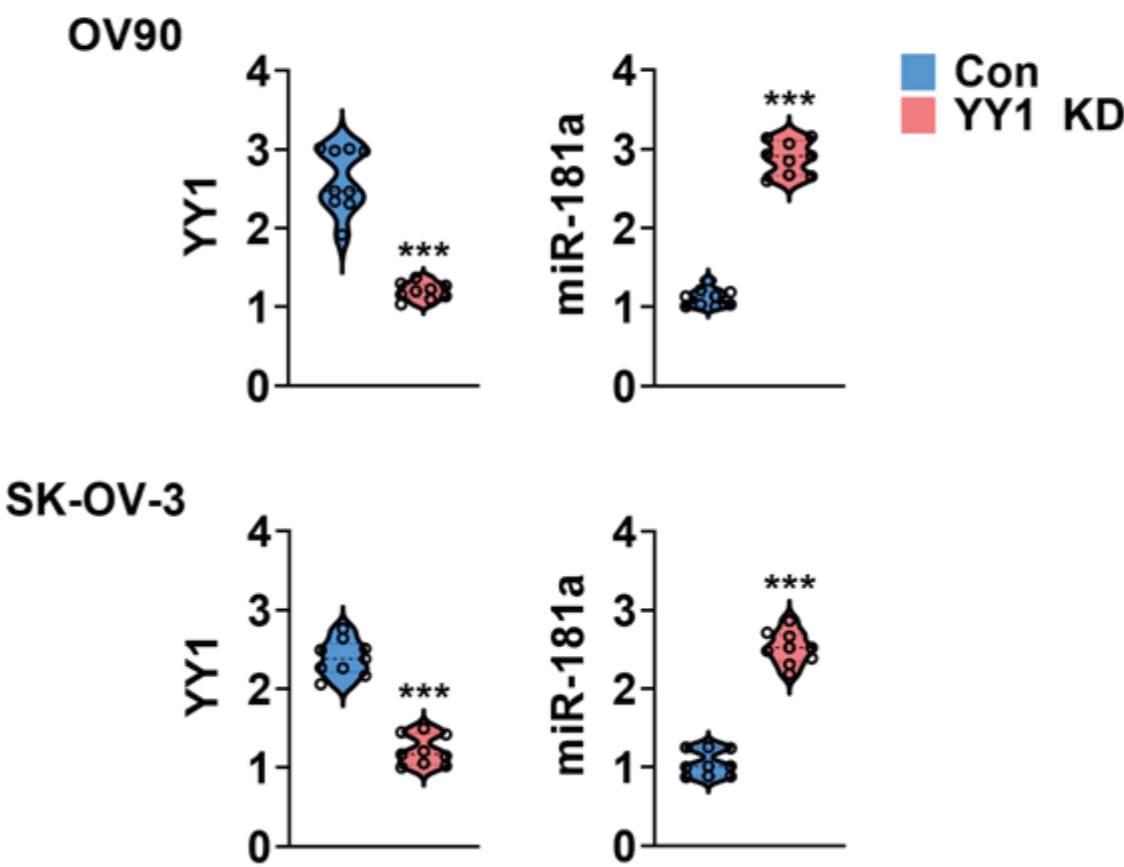

Supplement: Supplementary file 4 — Figure S4. Alteration of miR‐181a expression in ovarian cancer cells by YY1 knockdown. miR‐181a expression was analysed in the OV90 and SK‐OV‐3 cells transfected with control or YY1 shRNA using real‐time PCR. [file CPR-57-e13582-s002.pdf]

Supplementary Figure S5.

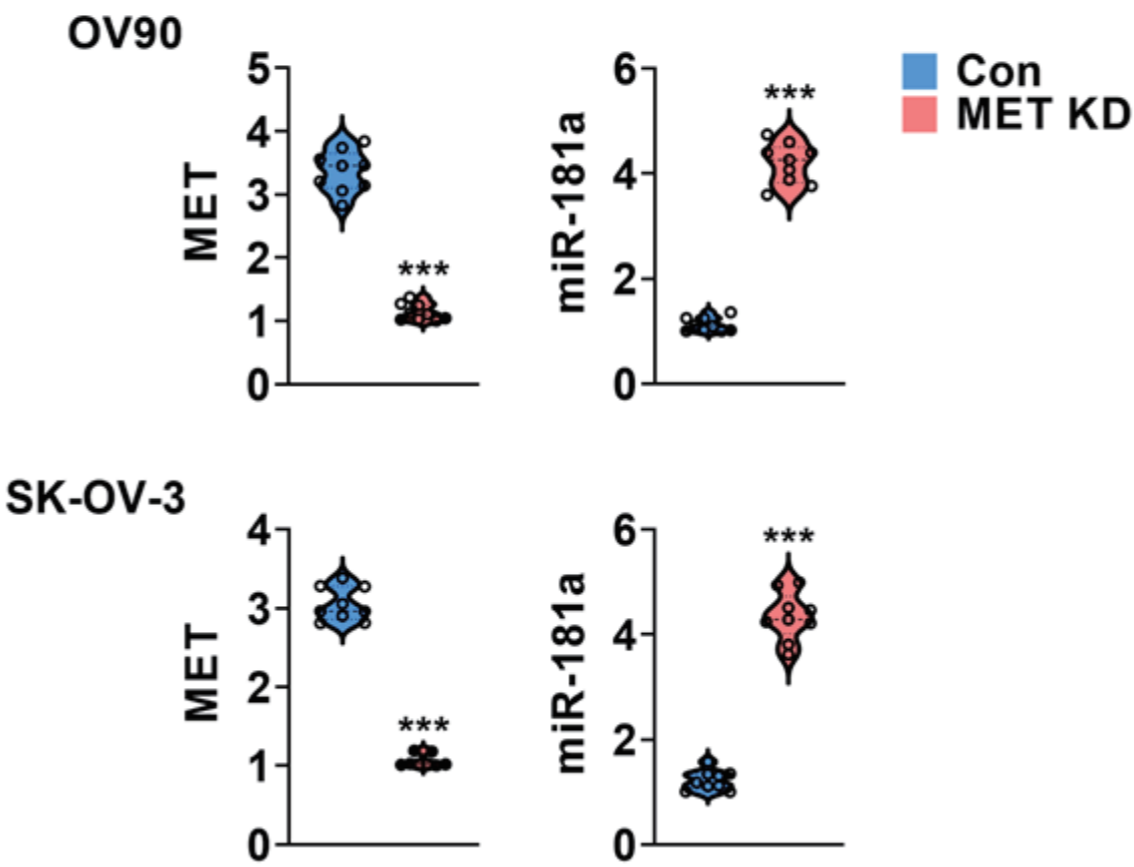

Supplement: Supplementary file 5 — Figure S5. Alteration of miR‐181a expression in ovarian cancer cells by MET knockdown. miR‐181a expression was analysed in the OV90 and SK‐OV‐3 cells transfected with control or MET shRNA using real‐time PCR. [file CPR-57-e13582-s007.pdf]
