# Supplementary material for: MET overexpression in ovarian cancer via CD24‐induced downregulation of miR‐181a: A signalling for cellular quiescence‐like state and chemoresistance in ovarian CSCs
Source: Cell Prolif. 2023 Nov 29;57(5):e13582. doi: 10.1111/cpr.13582 (PMC11056702; doi:10.1111/cpr.13582)
Supplement: Supplementary file 6 — Table S1. Primers for gene expression analysis and ChIP enrichment analysis. [file CPR-57-e13582-s009.docx]

**Supplementary Table S1. Primers for gene expression analysis and ChIP enrichment analysis.**

|  | Gene | Strand | Sequence (5’ to 3’) |
| --- | --- | --- | --- |
| Gene expression | CD24 | Forward | GCTCCTACCCACGCAGATTT |
|  |  | Reverse | CCACGAAGAGACTGGCTGTT |
|  | miR-181a | Forward | AACATTCAACGCTGTCGGTGAGT |
|  | MET | Forward | ACAACCCGAATACTGCCCAG |
|  |  | Reverse | GCTGATATCCGGGACACCAG |
|  | GAPDH | Forward | TGCACCACCAACTGCTTAGC |
|  |  | Reverse | GGCATGGACTGTGGTCATGAG |
|  | HPRT1 | Forward | TGACACTGGCAAAACAATGCA |
|  |  | Reverse | GGTCCTTTTCACCAGCAAGCT |
|  | SDHA | Forward | TGGGAACAAGAGGGCATCTG |
|  |  | Reverse | CCACCACTGCATCAAATTCATG |
|  | URT | Reverse | AACGAGACGACGACAGACTTTTTTTTTTTTT |
|  | Universal PCR | Reverse | AACGAGACGACGACAGACTTT |
| ChIP enrichment | MIR181A1 site 1 | Forward | CACCAACTTGGAATTCTGGA |
|  |  | Reverse | TCCATCTTTATTTTCACAACTTAATCC |
|  | MIR181A1 site 2 | Forward | TGGAAAAGATATAGGATTGGAAGG |
|  |  | Reverse | TCGAGCAATACAGAAAAATCG |
|  | MIR181A2 site 1 | Forward | GGAAAACAAGCAATTTTATTTATTAAC |
|  |  | Reverse | CCTCTCCATTTCTTTGCTTCC |
