# Supplementary material for: MET overexpression in ovarian cancer via CD24‐induced downregulation of miR‐181a: A signalling for cellular quiescence‐like state and chemoresistance in ovarian CSCs
Source: Cell Prolif. 2023 Nov 29;57(5):e13582. doi: 10.1111/cpr.13582 (PMC11056702; doi:10.1111/cpr.13582)
Supplement: Supplementary file 7 — Table S2. Clinical characteristics of the 64 patients with ovarian serous papillary carcinoma. [file CPR-57-e13582-s006.docx]

**Supplementary Table S2. Clinical characteristics of the 64 patients with ovarian serous papillary carcinoma.**

| Parameters | Mean ± SD / Range | No. of cases (N=64) |
| --- | --- | --- |
| Age (years) | 50.1 ± 11.6 / 20-75 |  |
| Mass size (cm) | 9.8 ± 5.3 / 2-30 |  |
| Stage |  |  |
| I |  | 0 |
| II |  | 11 |
| III |  | 49 |
| IV |  | 4 |
| CA19-9 (U/m) | 55.1 ± 182.7 / 0.5-1170 |  |
| CEA (ng/ml) | 1.5 ± 1.5 / 0.2-11.2 |  |
| Tumor recurrence |  | 53 |
| Patient deaths |  | 47 |
| Follow-up duration (months) | 81.2 ± 55.3/ 8-219 |  |

※ SD: standard deviation
