# Supplementary material for: MET overexpression in ovarian cancer via CD24‐induced downregulation of miR‐181a: A signalling for cellular quiescence‐like state and chemoresistance in ovarian CSCs
Source: Cell Prolif. 2023 Nov 29;57(5):e13582. doi: 10.1111/cpr.13582 (PMC11056702; doi:10.1111/cpr.13582)
Supplement: Supplementary file 8 — Table S3. Cox regression analysis for factors affecting disease‐free survival. [file CPR-57-e13582-s005.docx]

**Supplementary Table S3. Cox regression analysis for factors affecting disease-free survival.**

| Factors | Disease-free survival | |
| --- | --- | --- |
|  | HR (95% CI) | p-value |
| Age | 1.002 (0.971-1.035) | 0.8785 |
| Mass size | 0.961 (0.889-1.038) | 0.3123 |
| CA19-9 | 1.000 (0.996-1.004) | 0.9354 |
| CEA | 0.858 (0.509-1.447) | 0.5659 |
| CD24 volume | 1.02 (1.004-1.035) | 0.0119* |
| CD24 (volume*intensity) | 1.008 (1.002-1.013) | 0.0048* |
| MET volume | 1.002 (1.006-1.035) | 0.0062* |
| MET (volume*intensity) | 1.007 (1.002-1.011) | 0.0057* |
| CD24 modified score |  | 0.0387* |
| negative (0 and 1) | 1 |  |
| positive (2 and 3) | 3.558 (1.068-11.852) |  |
| MET modified score |  | 0.0459* |
| negative (0 and 1) | 1 |  |
| positive (2 and 3) | 2.951 (1.02-8.54) |  |
| CD24 MET modified score |  | 0.0464* |
| - / - | 1 |  |
| + / + | 7.705 (1.033-57.482) |  |

HR, hazard ratio; CI, confidence interval

* P-value < 0.05
