# Supplementary material for: MET overexpression in ovarian cancer via CD24‐induced downregulation of miR‐181a: A signalling for cellular quiescence‐like state and chemoresistance in ovarian CSCs
Source: Cell Prolif. 2023 Nov 29;57(5):e13582. doi: 10.1111/cpr.13582 (PMC11056702; doi:10.1111/cpr.13582)
Supplement: Supplementary file 9 — Data S1. Supporting information. [file CPR-57-e13582-s001.docx]

**Supplementary Information**

**Materials and Methods**

**Cell culture**

CD24-high and CD24-low clones, which were obtained from the mucinous cystadenocarcinoma tissue of a 46-year-old woman, were suspended in Iscove's modified Dulbecco's medium (IMDM, Gibco BRL, Grand Island, NY) as described previously (Oncogene. 2010;29(18):2672-2680). Caov-3 and SK-OV-3 were purchased from Korea Cell Line Bank (KCLB, Seoul, Korea). OV90 and HEK293T were purchased from the American Type Culture Collection (ATCC, Manassas, VA). Caov-3, OV90, and SK-OV-3 cells were maintained in Roswell Park Memorial Institute 1640 Medium (RPMI-1640, Gibco BRL, Grand Island, NY) supplemented with 10% heat-inactivated fetal bovine serum (FBS, Gibco BRL, Grand Island, NY) and 100 units/ml penicillin-streptomycin (Gibco BRL, Grand Island, NY) at 37°C in a humidified atmosphere of 5% CO2. HEK293T cells were maintained in Dulbecco's Modified Eagle Medium (DMEM, Gibco BRL, Grand Island, NY). The cell lines were authenticated by STR profiling, and no mycoplasma contamination was detected.

**Flow cytometry-based assays**

For the flow cytometry analysis, cells were subjected to immunostaining using anti-CD24 or anti-MET antibodies (BD Biosciences, East Rutherford, NJ). CD24-low and high cell populations were isolated by sorting the BD FACSAria™ cell sorter (BD Biosciences, East Rutherford, NJ). In the case of Caov3 cells, the lower 30% of the cells stained with CD24 antibody were sorted for CD24-low population, while the upper 30% were sorted for CD24-high population. The expression levels of CD24 and MET and cell cycle were analyzed using FACSCalibur™ flow cytometer (BD Biosciences, East Rutherford, NJ). For cell cycle analysis, cells were harvested, washed with PBS, and followed by fixation with 70% ethanol for 30 min. Next, the cells were washed with PBS, treated with ribonuclease (50 ul of 100 ug/ml), and added with PI (50 ug/ml).

**Gene manipulation**

CD24 and MET knockdown was performed with SureSilencing shRNA plasmids (Qiagen, Hilden, Germany), YY1 knockdown was performed using TRC shRNA (TRCN0000019894), and miR-181a overexpression was performed with LentimiRa-GFP-hsa-miR-181a-5p vector (Applied Biological Materials, Richmond, BC, Canada). Transfection was performed using Lipofectamine® LTX with Plus Reagent (Invitrogen, Carlsbad, CA).

**Luciferase reporter plasmid construction and dual luciferase assays**

The wild-type 3′UTRs of CD24 and MET containing predicted miR-181-binding sites were amplified by PCR using Caov-3 genomic DNA as a template. Mutant 3′UTR with deletions of seed sequence of each gene were generated by an overlap extension PCR method. Both wild-type and mutant 3′UTRs were inserted downstream of the firefly luciferase-coding gene at the XbaI site in the pGL3 control vector. The authenticity and orientation of the inserts were confirmed by sequencing. For luciferase assays, the transfection mixtures containing 200 ng of firefly luciferase reporter plasmid, 10 ng of pRL-TK (Renilla luciferase) (Promega, Madison, WI), and 500 ng of miR-181 plasmid were added into HEK293T cells in six-well plates using Lipofectamine® LTX with Plus Reagent (Invitrogen, Carlsbad, CA). The cells were harvested 48 hours after transfection, and luciferase activity was measured using a dual-luciferase reporter assay system (Promega, Madison, WI).

**Real-time PCR analysis**

Total RNA were extracted with Trizol (Invitrogen, Carlsbad, CA) and miRNeasy kit (Qiagen, Hilden, Germany) according to the manufacturer’s protocol. Total RNA was reverse-transcribed using HyperScriptTM RT Master Mix (GeneAll, Seoul, Korea). For miRNA cDNA synthesis, RNAs were first modified by the addition of a poly (A) tail using E. coli Poly (A) polymerase (E-PAP; New England Biolabs, Ipswich, MA) and then reverse-transcribed as previously described (Cell Cycle. 2006;5(17):1951-1956). For real-time PCR, 25 ng of the resulting cDNA were mixed with LaboPassTM SYBR Green Q Master (Cosmogenetch, Seoul, Korea) and primers (Supplementary Table S1) and then amplified in CFX Connect Real-Time System (Bio-Rad Laboratories, Hercules, CA). The expression of gene transcripts was normalized to the geomean of glyceraldehyde-3-phosphate dehydrogenase (GAPDH), Succinate Dehydrogenase Complex Flavoprotein Subunit A (SDHA), and Hypoxanthine Phosphoribosyltransferase 1 (HPRT1) expression, and then calculated according to the ΔΔCt method.

**Western blot analysis**

For sample preparation, cells were added with PRO-PREP Protein Extraction Solution (iNtRON Biotechnology, Seongnam-si, Korea), incubated at 4°C for 30 mins, and then centrifuged at 15,000 x g for 30 mins using Centrifuge 581R (Eppendorf, Hamburg, Germany). After protein quantification using Protein Assay Kit (Bio-Rad Laboratories, Hercules, CA), 20 ug of protein was subjected to electrophoresis on 10% polyacrylamide gels, transferred to a PVDF membrane (Millipore Corporation, Billerica, MA), and then probed with primary antibodies against CD24, MET, Src, phosphor Src (Y419), phosphor Src (Y529), YY1 (Abcam, Cambridge, UK), phospho YY-1 (S365, ThermoFisher Scientific, Waltham, MA), GAPDH (Santa Cruz Biotechnologies, Dallas, TX). Primary antibodies were captured with horseradish peroxidase (HRP)-conjugated secondary antibodies (GeneDepot, Katy, TX) and then visualized using WESTSAVE ECL Solution (AbFrontier, Seoul, Korea).

**Half maximal inhibitory concentration (IC50) determination**

Cells (5 x 10^3^ /well) were plated in 96-well plates (SPL Life Sciences, Pocheon-si, Korea). One day after seeding, cells were treated with cisplatin and carboplatin half-serially diluted from 100 uM. To measure viability, cells were incubated for 1 hour with 100 ul of serum-free RPMI containing 10 ul of Cell Counting Kit-8 solution (Dojindo Molecular Technologies, Inc, Rockville, MD). Absorbance at 450 and 650 nm was measured using a VersaMax Microplate Reader (Molecular Devices, San Jose, CA). IC50 was calculated using absorbance values (450 – 650 nm) and Prism (GraphPad Software, La Jolla, CA).

**Colony formation assay**

Cells were harvested using TrypLE TM Express enzyme (ThermoFisher Scientific, Waltham, MA), counted with LUNA-FX7 (Logos Biosystems, Gyeonggi-do, South Korea), plated (100 cells / well) onto 6-well plates (SPL Life Sciences, Pocheon-si, Korea), and then incubated at 37°C in a humidified atmosphere of 5% CO2. Colonies were fixed with 100% methanol for 20 min, incubated in crystal violet, and rinsed in water. The plates were inverted onto a tissue to dry overnight, and their images were captured for colony analysis using BX53 system microscope (Olympus, Tokyo, Japan).

**Graphical presentation**

Cluster heatmaps, Venn diagrams, Volcano plots, and Alluvial plots were plotted using SRPlot (<http://www.bioinformatics.com.cn/srplot>). Bar graphs and Symbols with connecting line graphs were plotted using Prism (GraphPad Software, La Jolla, CA). Matrix heatmap was plotted using the conditional formatting of Excel (Microsoft, Redmond, WA).

**Legends of Supplementary Figures and Tables**

**Supplementary Figure S1. The expression patterns of CD24 and MET in ovarian cancer patient tissues.**

A) Representative cases of CD24 expression assessed by the modified score 0 (a), 1 (b), 2 (c), and 3 (d). B) Intense staining of CD24 in the tumor cells located at the papillary tip. C) A frequent pattern of CD24 expression in the tumor cells invading stroma. D) Representative images of MET expression. (a) Diffuse staining of MET (the modified score 3), (b) MET staining in the tumor cells at the papillary tip, (c) A frequent pattern of MET expression in the tumor cells invading stroma.

**Supplementary Figure S2. CD24-upregulated expression of miRNAs in ovarian cancer cells.**

A) Venn diagram presentation of the miRNAs commonly upregulated in C4 and the CD24-high population of Caov-3 cells. Presentation of the miRNAs commonly upregulated in the CD24-high populations of primary ovarian cancer cells and Caov-3 cells using B) Scatter plot and C) Superimposed symbols with connecting line plot.

**Supplementary Figure S3. Plasmid constructs for binding assay.**

The wild and mutant plasmid constructs of A) CD24 and B) MET 3’ UTR

**Supplementary Figure S4. Alteration of miR-181a expression in ovarian cancer cells by YY1 knockdown.**

miR-181a expression was analyzed in the OV90 and SK-OV-3 cells transfected with control or YY1 shRNA using real-time PCR.

**Supplementary Figure S5. Alteration of miR-181a expression in ovarian cancer cells by MET knockdown.**

miR-181a expression was analyzed in the OV90 and SK-OV-3 cells transfected with control or MET shRNA using real-time PCR.

**Supplementary Table S1. Primers for gene expression analysis and ChIP enrichment analysis.**

**Supplementary Table S2. Clinical characteristics of the 64 patients with ovarian serous papillary carcinoma.**

**Supplementary Table S3. Cox regression analysis for factors affecting disease-free survival.**
